# Case report: Changes in serum bevacizumab concentration in a hemodialysis patient with unresectable colorectal cancer treated with FOLFIRI plus bevacizumab

**DOI:** 10.3389/fonc.2022.947013

**Published:** 2022-08-30

**Authors:** Toshimitsu Tanaka, Hiroyuki Suzuki, Tomoyuki Ushijima, Sachiko Nagasu, Yoshito Akagi, Takumi Kawaguchi, Keisuke Miwa

**Affiliations:** ^1^ Multidisciplinary Treatment Cancer Center, Kurume University Hospital, Kurume, Japan; ^2^ Division of Gastroenterology, Department of Medicine, Kurume University School of Medicine, Kurume, Japan; ^3^ Department of Surgery, Kurume University School of Medicine, Kurume, Japan

**Keywords:** hemodialysis, bevacizumab, concentration, chemotherapy, malignancy, folinic acid, 5-fluorouracil, irinotecan

## Abstract

The global incidence of colorectal cancer (CRC) in patients receiving hemodialysis is steadily rising. However, current information on the clinical use of chemotherapy for patients undergoing hemodialysis with CRC is limited. Herein, we describe a clinical course of a 74-year-old patient undergoing hemodialysis with unresectable CRC treated with folinic acid, 5-fluorouracil (5FU), and irinotecan (FOLFIRI) plus bevacizumab whose changes in serum bevacizumab concentration were analyzed. Treatment was initiated with a standard dosage of 5-FU and 80% of the standard dose of irinotecan to avoid any adverse events. However, neutropenia (grade 4) was observed after five treatment cycles, which prompted a dose reduction of 5-FU and irinotecan, after which treatment was safely completed. Progression-free survival of the patient was 7.5 months. Changes in serum bevacizumab concentration were similar to those documented in patients with normal renal function. In addition, no bevacizumab-related adverse events occurred. It was inferred that FOLFIRI plus bevacizumab therapy could be implemented as a safe and efficient treatment for patients undergoing hemodialysis with unresectable CRC. To the best of our knowledge, this is the first report of the analysis of serum bevacizumab concentrations in a patient undergoing hemodialysis with unresectable CRC.

## Introduction

Hemodialysis alleviates symptoms of patients with end-stage renal disease, and more than one million people currently benefit from long-term hemodialysis worldwide (1). This number is expected to increase owing to an aging global society and an increase in the prevalence of type 2 diabetes (2). It is believed that the incidence of malignancy in patients undergoing hemodialysis is higher due to mechanisms such as chronic oxidative stress, altered DNA repair, and compromised immune system (3). Therefore, it is expected that the number of patients with cancer receiving hemodialysis will increase in the future. However, existing knowledge regarding such cases is scarce, which makes cancer treatment challenging.

Colorectal cancer (CRC) is one of the most common types of malignancies of the digestive system (4). With the advent of chemotherapeutic combinations such as folinic acid, 5-fluorouracil (5FU), and irinotecan [FOLFIRI] or 5-FU, leucovorin, and oxaliplatin [FOLFOX] plus molecular targeted therapy (such as bevacizumab, aflibercept, ramucirumab, cetuximab, and panitumumab), the treatment of metastatic/unresectable CRC has significantly advanced (5). However, it is important to identify a dose that produces an effective antitumor effect without adverse events (AEs) to maintain long-term treatment and achieve favorable patient outcomes. Therefore, establishing an appropriate molecular targeted therapy for patients with cancer on chronic hemodialysis is crucial, as the drug pharmacokinetics in these patients differ from those of healthy individuals.

Herein, we report changes in the blood bevacizumab concentration of a patient with unresectable colon cancer, in whom FOLFIRI plus bevacizumab treatment was performed safely during hemodialysis.

## Case description

A 74-year-old Japanese man with end-stage renal failure, precipitated by an unknown primary disease, had been maintained on hemodialysis since 2018. In April 2019, he was diagnosed with advanced sigmoid colon cancer and underwent a colectomy for the same. The pathological stage of the tumor was confirmed as IIIC (T4aN2M0). Although the patient was informed that there was a high chance of cancer recurrence, he did not wish to receive adjuvant therapy. Follow-up computed tomography scan performed in December 2019 revealed multiple lung and liver metastases, considered to be recurrent colon cancers. The molecular biology of the tumor was as follows; microsatellite instability, MSS; *KRAS* mutation, exon 2 (G12D); *BRAF* mutation, wild type; *UGT1A1* mutation, absent (**Table 1**). Thereafter, the patient was treated with modified FOLFOX6 as first-line chemotherapy in April 2020. Unfortunately, the liver metastases increased after 21 cycles of treatment, and in April 2021, the patient was referred to our hospital for further treatment. Physical and blood examinations revealed no notable changes, and his performance status was 0 at admission. Laboratory data obtained on admission to our hospital and the genetic information of the cancer are shown in **Table 1**.

**Table 1:** Laboratory data on admission.

Blood biochemistry		Peripheral blood	
TP	6.1 g/dL	WBC	7000/μL
Albumin	3.4 g/dL	Neutrophil	74.9%
T-Bil	0.4 mg/dL	Lymphocyte	16.9%
AST	16 IU/L	Monocyte	4.9%
ALT	12 IU/L	Eosinophil	2.7%
LDH	188 IU/L	Basophil	0.6%
ALP	84 IU/L	RBC	364 × 10^4^/μL
γ-GTP	34 IU/L	Hb	12.1 g/dL
BUN	83 mg/dL	Ht	37.2%
Cre	10.42 mg/dL	Plt	19.7 × 10^4^/μL
UA	8.9 mg/L	**Viral markers**	
Na	143 mEq/L	HBsAg	(-)
K	3.7 mEq/L	Anti-HBc	(-)
Cl	107 mEq/L	Anti-HCV	(-)
Ca	8.7 mg/dL	**Tumor markers**	
CRP	0.29 mg/dL	CEA	85.5 ng/mL
**Coagulation**		CA19-9	115.0 U/mL
PT%	69%	**Tumor-related gene status**	
PT-INR	1.27	MSI	MSS
		*KRAS* mutation	exon 2 (G12D)
		*BRAF* mutation	wild type
		*UGT1A1* mutation	absent

TP, total protein; T-Bil, total bilirubin; AST, aspartate aminotransferase; ALT, alanine aminotransferase; LDH, lactate dehydrogenase; ALP, alkaline phosphatase; γ-GTP, γ-glutamyl transpeptidase; BUN, blood urea nitrogen; Cre, creatinine; UA, uric acid; Na, sodium; K, potassium; Cl, chloride; Ca, calcium; CRP, C-reactive protein; WBC, white blood cell; RBC, red blood cell; Hb, hemoglobin; Ht, hematocrit; Plt, platelet count; PT, prothrombin time; INR, international normalized ratio; HbsAg, hepatis B surface antigen; Anti-HBc, hepatitis B core antibody; Anti-HCV, hepatitis C virus antibody; CEA, carcinoembryonic antigen; CA19-9, carbohydrate antigen 19-9; MSI, microsatellite instability.

The body surface area of the patient was 1.46 m^2^, and he was treated with FOLFIRI [irinotecan 120 mg/m^2^ intravenously infused over 120 min (80% of the standard dose in Japan), with levofolinate 200 mg/m^2^ over 120 min, followed by 5-FU 2400 mg/m^2^ intravenously infuse over 46 h (without 5-FU bolus)] plus bevacizumab (5 mg/kg intravenously infused over 30 min) every 2 weeks. Hemodialysis was performed on the second day for convenience and scheduling following FOLFIRI therapy and repeated 3 times per week. To measure serum bevacizumab concentrations, blood samples were collected at five time points: pretreatment (defined as day 0), before hemodialysis on day 2, day 4, day 6, and day 8. For days 4, 6, and 8, blood samples were obtained just before the start of dialysis. All blood samples were centrifuged immediately after collection at 3000 rpm for 10 min, and the obtained serum was further centrifuged at 3000 rpm for 20 min. The centrifuged samples were stored in a freezer until measurement using a bevacizumab ELISA Kit (#ab237642; Abcam, Cambridge, UK).

**Figure 1:**
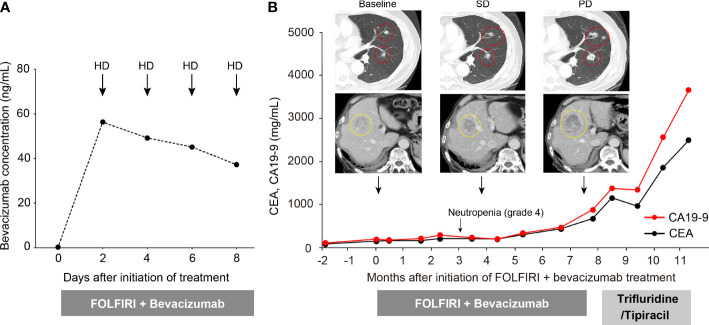
Changes in serum bevacizumab concentration and clinical course of the patient. **(A)** Changes in serum bevacizumab concentration at five time points after treatment initiation. **(B)** Clinical course of the patient. Red and yellow circles represent lung and liver metastases, respectively.

It was observed that the serum concentration of bevacizumab increased to 56.3 µg/mL on day 2 but then decreased over time regardless of hemodialysis; the serum concentration on day 8 decreased to 37.1 µg/mL (**Figure 1A**). At the fifth cycle after treatment initiation, neutropenia (grade 4) was observed, and thus we reduced the dosage of 5-FU to 2000 mg/m^2^ and that of irinotecan to 100 mg/m^2^. However, no bevacizumab-related AEs were observed. Regarding therapeutic effects, stable disease was maintained for 7.5 months under treatment with FOLFIRI plus bevacizumab. Thereafter, the disease showed progression with elevated tumor markers and increased size and number of lung and liver metastases (**Figure 1B**), following which the treatment regimen was changed to trifluridine/tipiracil. Unfortunately, the therapeutic effect was poor, and the treatment was transitioned from chemotherapy to best supportive care.

## Discussion

In recent years, the number of patients undergoing hemodialysis with malignancies has increased. According to a prospective study that followed 454 patients undergoing dialysis, 12% of the reported deaths were attributable to malignancies (6). Research conducted in Australia on a large cohort of patients with end-stage renal disease revealed that the incidence of all types of malignancies in patients undergoing dialysis was higher than that in patients not undergoing dialysis (standardized mortality ratio 1.35; 95% CI, 1.27–1.45) (7). It has been reported that the risk of carcinogenesis is increased in patients undergoing dialysis due to increased genetic damage caused not only by chronic oxidative stress but also from dysbiosis and a weakened immune system, which enhances the production of inflammatory mediators such as tumor necrosis factor-α and interleukin-6 (8, 9).

There are no established chemotherapy regimens for patients undergoing hemodialysis with malignancies, and the optimal dosage remains unclear. A review of expert opinions on cancer risk and the use of chemotherapeutic agents in patients with end-stage renal failure recommends several regimens of chemotherapy for patients undergoing dialysis. The use of chemotherapeutic agents has been reported to be safe in patients with end-stage renal disease, while appropriate dose adjustments should be considered based on the dialysability and pharmacokinetics of the drug (10). The safety of 5-FU in patients undergoing dialysis has been widely studied, and it has been shown that approximately 80% of 5-FU is inactivated by hepatic dihydropyridine dehydrogenase; thus, it is considered that dose reduction is not necessary for these patients (11). Following intravenous administration, irinotecan, a camptothecin that inhibits topoisomerase I, is converted into several metabolites, including the active metabolite 7-ethyl-10 hydroxycamptothecin (SN-38) (12). In patients undergoing dialysis, irinotecan is minimally dialyzable, and the active metabolite SN-38 is nondialyzable (13, 14). Therefore, lower creatinine clearance (35–66 mL/min) is associated with a significantly increased risk of irinotecan-induced neutropenia (15). Irinotecan administration in patients undergoing hemodialysis with unresectable CRC has been reported to cause fatal febrile neutropenia and diarrhea, requiring a dose reduction (16–18). For the above reasons, we started with the standard dosage of 5-FU without a bolus dose and 80% of the standard dose of irinotecan to avoid associated AEs. However, in the current case, neutropenia (grade 4) was observed after five treatment cycles, which prompted a dose reduction of 5-FU and irinotecan, after which the treatment was safely continued; no AEs were encountered subsequently.

Bevacizumab, a monoclonal antibody that inhibits vascular endothelial growth factor, is extensively used for cancer treatment (19). In patients with CRC, the prognostic effect of adding bevacizumab to chemotherapy was previously demonstrated in clinical trials (CALGB trial, FIRE.3 trial, and ML18147 study) (20–22). In the current case, progression-free survival was 7.5 months. This was similar to a previous report that indicated a progression-free survival of 6.1 months in patients with unresectable CRC treated with FOLFIRI plus bevacizumab therapy as second-line treatment (23). Therefore, it seems that a relatively good outcome was obtained even though the molecular weight of bevacizumab is 149,000 Da, which is too large to be removed by dialysis, and the patient in the present case was under hemodialysis. Our results were consistent with those of a study that evaluated the pharmacokinetics of bevacizumab in a patient undergoing dialysis with metastatic CRC (24) and revealed similar pharmacokinetic parameters in patients undergoing dialysis as the reference values in patients with normal renal function. Furthermore, changes in the blood concentrations of bevacizumab in the present case were similar to those stated in the package insert of bevacizumab (Avastin^®^; Chugai Pharmaceutical Co. Ltd., Tokyo) marketed in Japan, for a single dose of 5 mg/kg in patients with CRC. No bevacizumab-related AEs occurred in this patient; therefore, the undertaken treatment regimen can be assumed to be safe and feasible.

To the best of our knowledge, this is the first clinical case report revealing that changes in the serum concentrations of bevacizumab in patients undergoing dialysis are similar to those in patients with normal renal function. However, this report has several limitations. This report is just one case, and the concentration of bevacizumab was evaluated in only one cycle, which is not sufficient to assess the efficacy and tolerability of chemotherapy in dialysis patients. Therefore, evaluation in multiple cycles and case accumulation is needed in the future.

## Conclusion

FOLFIRI plus bevacizumab therapy for patients undergoing hemodialysis with unresectable CRC could be a safe and effective treatment. This is the first clinical case to report that changes in the serum concentrations of bevacizumab in patients undergoing dialysis are similar to those in patients with normal renal function.

## Data availability statement

The original contributions presented in the study are included in the article/supplementary materials, further inquiries can be directed to the corresponding author.

## Ethics statement

Ethical review and approval was not required for the study on human participants in accordance with the local legislation and institutional requirements. The patients/participants provided their written informed consent to participate in this study.

Written informed consent was obtained from the individual(s) for the publication of any potentially identifiable images or data included in this article.

## Author contributions

TT and HS wrote the manuscript. TU and SN participated in the acquisition of data and critical revision. YA, TK, KM supervised the study. All authors contributed to the article and approved the submitted version.

## Acknowledgments

We would like to thank Editage (www.editage.com) for English language editing.

## Conflict of interest

The authors declare that the research was conducted in the absence of any commercial or financial relationships that could be construed as a potential conflict of interest.

## Publisher’s note

All claims expressed in this article are solely those of the authors and do not necessarily represent those of their affiliated organizations, or those of the publisher, the editors and the reviewers. Any product that may be evaluated in this article, or claim that may be made by its manufacturer, is not guaranteed or endorsed by the publisher.

## Abbreviations

FOLFIRI, folinic acid, fluorouracil and irinotecan; HD, hemodialysis; CEA, carcinoembryonic antigen; CA19-9, carbohydrate antigen 19-9; SD, stable disease; PD, progressive disease.
